# MTHFR C677T and MTR A2756G polymorphisms and the homocysteine lowering efficacy of different doses of folic acid in hypertensive Chinese adults

**DOI:** 10.1186/1475-2891-11-2

**Published:** 2012-01-10

**Authors:** Xianhui Qin, Jianping Li, Yimin Cui, Zeyuan Liu, Zhigang Zhao, Junbo Ge, Deming Guan, Jian Hu, Yanni Wang, Fumin Zhang, Xin Xu, Xiaobin Wang, Xiping Xu, Yong Huo

**Affiliations:** 1 Institute of Biomedicine, Anhui Medical University, Hefei, China; 2 Department of Cardiology and Heart Center, Peking University First Hospital, Beijing, China; 3 Department of Pharmacy, Peking University First Hospital, Beijing, China; 4 Department of Pharmacy, First Affiliated Hospital of Academy of Military Medical, Sciences of China, Beijing, China; 5 Department of Pharmacy, Beijing Tiantan Hospital, Capital Medical University, Beijing, China; 6 Department of Cardiology, Zhongshan Hospital Fudan University, Shanghai, China; 7 Department of Cardiology, First Clinical Medical College of Harbin Medical University, Harbin, China; 8 Department of Cardiology, First Affiliated Hospital of China Medical University, Shenyang, China; 9 Department of Cardiology, First Affiliated Hospital of the School of Medicine, Xi'an Jiaotong University, Xi'an, China; 10 Department of Cardiology, First Affiliated Hospital of Nanjing Medical University, Nanjing, China; 11 Guangdong Institute of Nephrology, Southern Medical University, Guangzhou, China; 12 Mary Ann and J. Milburn Smith Child Health Research Program, Children's Memorial Hospital and Children's Memorial Research Center, Department of Pediatrics, Northwestern University Feinberg School of Medicine, Chicago, USA

**Keywords:** Folic acid supplementation, MTHFR C677T polymorphism, MTR A2756G polymorphism, Homocysteine-lowering efficacy

## Abstract

**Background:**

This study aimed to investigate if the homocysteine-lowering efficacy of two commonly used physiological doses (0.4 mg/d and 0.8 mg/d) of folic acid (FA) can be modified by individual methylenetetrahydrofolate reductase (MTHFR) C677T and/or methionine synthase (MTR) A2756G polymorphisms in hypertensive Chinese adults.

**Methods:**

A total of 480 subjects with mild or moderate essential hypertension were randomly assigned to three treatment groups: 1) enalapril only (10 mg, control group); 2) enalapril-FA tablet [10:0.4 mg (10 mg enalapril combined with 0.4 mg of FA), low FA group]; and 3) enalapril-FA tablet (10:0.8 mg, high FA group), once daily for 8 weeks.

**Results:**

After 4 or 8 weeks of treatment, homocysteine concentrations were reduced across all genotypes and FA dosage groups, except in subjects with MTR 2756AG /GG genotype in the low FA group at week 4. However, compared to subjects with MTHFR 677CC genotype, homocysteine concentrations remained higher in subjects with CT or TT genotype in the low FA group (*P *< 0.05 for either of these genotypes) and TT genotype in the high FA group (*P *< 0.05). Furthermore, subjects with TT genotype showed a greater homocysteine-lowering response than did subjects with CC genotype in the high FA group (mean percent reduction of homocysteine at week 8: CC 10.8% vs. TT: 22.0%, *P *= 0.005), but not in the low FA group (CC 9.9% vs. TT 11.2%, *P *= 0.989).

**Conclusions:**

This study demonstrated that MTHFR C677T polymorphism can not only affect homocysteine concentration at baseline and post-FA treatment, but also can modify therapeutic responses to various dosages of FA supplementation.

## Background

Traditional risk factors are estimated to account for only part of cardiovascular disease (CVD) risk [1]. Non-traditional risk factors such as increased homocysteine concentrations are believed to be causally related to CVD [2]. The interactive effect between hypertension and hyperhomocysteinemia on the risk of CVD has received great attention [3]. Our previous meta-analysis [4] suggested that folic acid (FA) supplementation could significantly reduce the risk of stroke by 18% [Relative Risk (RR):0.82, 95% Confidence Interval (CI): 0.68-1.00; *P *= 0.045), and an even greater beneficial effect was seen in those trials with no or partial FA fortification (RR: 0.75, 95%CI: 0.62-0.91; *P *= 0.003). Furthermore, FA supplementation was found to significantly reduce CVD risk in patients with end stage renal disease or advanced chronic kidney disease (creatinine clearance < 30 mL/min) by 15% (RR: 0.85; 95CI: 0.76-0.96, P = 0.009), particularly in trials with no or partial FA fortification (RR: 0.80; 95% CI: 0.65-0.99; p = 0.04) [5]. These findings underscore the importance of effectively lowering homocysteine concentration in the prevention of CVD, particularly in populations with a high prevalence of hypertension and hyperhomocystienemia but without FA fortification.

Methylenetetrahydrofolate reductase (MTHFR) and methionine synthase (MTR) are the main regulatory enzymes for homocysteine metabolism. MTHFR converts 5, 10-methylene-THF into 5-methyl-THF. Polymorphism of MTHFR C677T leads to a reduction in enzyme activity, which may lead to an increased concentration of plasma homocysteine and lower levels of serum folate, particularly in those with low folate intake [6]. MTR catalyzes the demethylation of 5-methyl-THF to THF and the remethylation (using the methyl group donated by 5-methyl-THF) of homocysteine to methionine. A common polymorphism in the MTR gene (A2756G) also seems to influence plasma homocysteine [7].

To our knowledge, no published trial has investigated if the homocysteine-lowering efficacy of two commonly used doses (0.4 mg/d and 0.8 mg/d) of FA can be modified by individual MTHFR C677T and MTG A2756G polymorphisms in hypertensive adults without FA fortification. In this report, we analyzed the data from a randomized, double-blind, controlled trial that included three intervention groups: 1) enalapril only (10 mg, control group); 2) enalapril-FA tablet (10 mg enalapril combined with 0.4 mg of FA, low FA group); and 3) enalapril-FA tablet (10 mg enalapril combined with 0.8 mg of FA, high FA group), once daily for 8 weeks. We sought to assess if MTHFR or MTR genotypes can influence a change in plasma homocysteine concentration in response to each of the two different dosages of FA supplementation.

## Methods

This was a multicenter, randomized, double-blind controlled trial in hypertensive Chinese adults (clinicaltrials.gov; identifier: NCT00520247) [8]. The details regarding "Subjects", "Intervention and data collection", "Laboratory methods", and "Statistical Analysis" have been previously described [9]. Briefly, 480 patients with mild or moderate hypertension were recruited from six hospitals in different Chinese regions (Ha'erbin, Shanghai, Shenyang, Beijing, Xi'an, and Nanjing) from September-December 2005. Eligible subjects were randomly and double-blindly assigned to one of the three treatment groups: 1) enalapril tablet (10 mg, control group); 2) enalapril-FA tablet (10 mg enalapril combined with 0.4 mg of FA, low FA group); or 3) enalapril-FA tablet (10 mg enalapril combined with 0.8 mg of FA, high FA group), once daily for 8 weeks.

Demographic and clinical information were obtained at baseline. Blood homocysteine concentrations were examined at baseline and at 4 and 8 weeks of the trial. MTHFR C677T and MTR A2756G genotypes were determined for each study subject.

All analyses were performed according to the principle of intention to treat. Homocysteine and folate concentrations in the natural logarithms (due to their positively skewed distributions) were analyzed as continuous variables. The relative change of homocysteine is expressed as the change of homocysteine after FA treatment relative to baseline concentration [(homocysteine after treatment-homocysteine at baseline)/(homocysteine at baseline)], which was applied as the primary outcome. Multivariable regression models were applied to compare the relative change of homocysteine among different groups. The additive models were used to test the gene-gene interactive effect between the MTHFR C677T and the MTR A2756G polymorphisms on the homocysteine-lowering efficacy of FA supplementation with and without adjustments for the potential non-genetic confounding factors. Statistical analysis was performed using SAS software version 6.12 (SAS Institute, Cary, NC, USA).

## Results

A total of 480 patients were recruited for this study. This analysis excluded 35 subjects who were ineligible to participate in the trial (7 in control group: 9 in low FA group; 8 in high FA group) or without data of MTHFR genotype (2 in high FA group) or MTR A2756G (1 in control group; 4 in low FA group; 4 in high FA group). Accordingly, a total of 445 subjects were included in our final analysis.

The prevalence of MTHFR C677T polymorphisms 677 CC, 677 CT, and 677TT were 0.256, 0.497, and 0.247, respectively. The prevalence of the 2756AA, 2756AG, and 2756 GG genotypes were 0.820, 0.171, and 0.009, respectively. This population had no significant deviations in genotype distributions from expected Hardy-Weinberg equilibrium. Because there were only four subjects homozygous for the G2756 allele, the genotypes AG and GG were combined for statistical analyses.

Table 1 shows the baseline characteristics of the subjects according to MTHFR and MTR genotypes. Subjects with MTHFR 677 TT genotype had significantly higher homocysteine concentrations (median: 17.5 μmol/L) than did those with CT (11.5 μmol/L) or CC (11.1 μmol/L) genotype (P < 0.01 for either of these genotypes). However, homocysteine concentrations did not differ among subjects with different genotypes of the MTR gene. There was no significant interactive effect between MTHFR C677T and MTR A2756G polymorphisms on homocysteine concentrations at baseline.

**Table 1:** Baseline characteristics of participants by MTHFR C677T or MTR A2756G polymorphism*^1^*

		MTHFR C677T				MTR A2756G		
	All	CC	CT	TT	*P*	AA	AG/GG	*P*
N	445	114	221	110		365	80	
Sex(Male), no.(%)	191(42.9)	51(44.7)	91(41.2)	49(44.5)	0.761	155(42.5)	36(45.0)	0.678
Age, yrs	57.0(9.8)	57.0(9.5)	56.4(9.6)	58.2(10.4)	0.284	57.0(9.8)	57.0(9.9)	0.971
BMI, kg/m^2^	25.7(3.5)	25.5(3.9)	25.8(3.3)	25.7(3.3)	0.758	25.8(3.5)	25.2(3.5)	0.137
SBP, mmHg	154.0(11.2)	154.0(11.5)	153.8(11.4)	154.5(10.5)	0.863	153.8(11.2)	155.1(11.2)	0.324
DBP, mmHg	93.0(8.3)	92.4(8.5)	93.4(7.8)	92.8(9.0)	0.530	93.3(8.0)	91.4(9.4)	0.052
FPG, mmol/L	5.5(1.3)	5.5(1.5)	5.4(1.2)	5.7(1.2)	0.312	5.5(1.3)	5.5(1.2)	0.865
TC, mmol/L	5.0(1.1)	5.0(1.4)	4.9(1.0)	5.1(1.0)	0.555	5.0(1.1)	4.9(1.1)	0.259
HDL-C, mmol/L	1.3(0.4)	1.3(0.4)	1.3(0.4)	1.3(0.4)	0.479	1.3(0.4)	1.3(0.3)	0.542
TG, mmol/L	1.7(1.2)	1.7(1.0)	1.8(1.2)	1.7(1.5)	0.970	1.8(1.3)	1.6(0.8)	0.252
Creatinine, μmol/L	69.5(17.5)	68.0(17.5)	69.2(17.3)	71.9(17.9)	0.233	69.2(17.9)	71.3(15.6)	0.334
Folate, nmol/L*^2^*	12.6(1.5)[12.3]	13.7(1.4)[13.4]	13.0(1.5)[12.6]	11.0(1.4)[10.8] *	< 0.001	12.8(1.5)[12.5]	11.9(1.5)[11.6]	0.107
Homocysteine, μmol/L*^2^*	13.2(1.6)[12.1]	11.2(1.4)[11.1]	12.0(1.4)[11.5]	18.8 (1.8)[17.5] *	< 0.001	13.1(1.6)[12.1]	13.3(1.6)[12.6]	0.794

BMI = body mass index, SBP = systolic blood pressure, DBP = diastolic blood pressure, FPG = fasting plasma glucose,

TC = total cholesterol, HDL-C = high-density lipoprotein cholesterol, TG = triglyceride;

*^1^*Means (SD); *^2^*geometric mean (anti-log SD), median in brackets; * Significantly different from the CT or CC genotype, *P *< 0.001.

Compared to baseline, after 4 or 8 weeks of FA treatment decreases in plasma homocysteine concentrations were seen across all MTHFR C677T genotypes and FA dosage groups (Table 2). Even after the treatment, homocysteine concentrations in subjects with TT and CT genotypes remained significantly higher at week 4 or week 8 (*P *< 0.05 for either of these genotypes) compared to those with CC genotype in the low FA group. In contrast, only subjects with TT genotype had higher homocysteine concentrations compared to CC genotype (*P *< 0.05) at week 4 or week 8 in the high FA group (Table 2).

**Table 2:** Response of plasma homocysteine to different doses and duration of folic acid supplementation by MTHFR C677T genotypes

	N	Homocysteine, μmol/L*^1^* Percent relative change of homocysteine*^2^*			Difference in change of Homocysteine at 4 weeks by genotypes*^3^*			Difference in change of Homocysteine at 8 weeks by genotypes*^3^*		
	
		baseline	4 weeks	8 weeks	*β*	*SE*	*P*	*β*	*SE*	*P*
Control										
CC	41	11.6(1.4)[11.3]	11.9(1.3)[11.8]	11.8(1.4)[11.3]						
			3.35(13.7)	2.2(14.0)	ref			ref		.
CT	73	11.0(1.4)[11.1]	11.2(1.4)[11.3]	11.3(1.4)[11.1]						
			2.25(13.2)	3.1(14.0)	-3.69	2.94	0.209	-1.80	3.45	0.601
TT	38	18.1(1.9)[14.7] ^#^	18.0(1.9)[15.6] ^#^	18.1(1.8)[16.5] ^#^						
			1.19(19.7)	3.3(27.0)	-4.05	3.33	0.225	-0.52	3.91	0.895
Total	152	12.7(1.6)[12.0]	12.8(1.6)[11.7]	12.8(1.6)[12.0]						
			2.3(15.1)	2.9(18.0)						
Low FA										
CC	34	11.0(1.3)[11.0]	9.9(1.3)[9.4]*	9.8(1.3)[9.5] *						
			-8.8(13.0)	-9.9(14.9)	ref			ref		
CT	79	12.6(1.5)[11.4]	11.5(1.4)[11.1] *^#^	11.2(1.4)[10.7] *^#$^						
			-7.6(15.4)	-9.7(16.8)	2.14	3.35	0.523	2.52	3.88	0.516
TT	34	17.5(1.8)[17.2] ^#^	15.2(1.7)[14.2] *^#^	14.8(1.6)[14.2] *^#^						
			-10.6(21.3)	-11.2(27.1)	-1.16	3.95	0.769	0.06	4.58	0.989
Total	147	13.2(1.6)[11.6]	11.8(1.5)[11.3] *	11.6(1.5)[10.8] *^$%^						
			-8.6(16.4) ^%^	-10.1(19.1) ^%^						
High FA										
CC	39	11.0(1.4)[10.8]	10.2(1.4)[10.1] *	9.7(1.4)[9.8] *^$^						
			-5.9(14.2)	-10.8(11.1)	ref			ref		
CT	69	12.3(1.3)[12.0]	10.8(1.3)[11.0] *	10.7(1.3)[10.5] *						
			-11.2(14.5)	-11.2(16.5)	-6.21	3.19	0.052	-0.47	3.39	0.890
TT	38	20.8(1.8)[19.6] ^#^	15.8(1.6)[14.2] *^#^	15.4(1.6)[14.1] *^#^						
			-20.3(22.2) ^#^	-22.0(23.8) ^#^	-15.51	3.72	< 0.001	-11.12	3.95	0.005
Total	146	13.7(1.6)[12.7]	11.7(1.5)[11.4] *	11.5(1.5)[10.8] *^%^						
			-12.1(17.5) ^%^	-13.9(18.1) ^%^						

*^1^*Geometric mean (anti-log SD), median in brackets;

*^2^*[(Homocysteine after treatment - homocysteine at baseline)/(homocysteine at baseline)]*100, Mean(SD);

*^3^*Regression model was adjusted for age, sex, BMI, baseline SBP, DBP, creatinine, TG, HDL-C, TC, FPG, folate, folate change, MTR A2756G polymorphism and study centers;

*Significantly different from baseline, *P *< 0.05; ^$^Significantly different from week 4, *P *< 0.05;

^#^Significantly different from the CC genotype, *P *< 0.05; ^%^Significantly different from control group, *P *< 0.05.

Furthermore, in the high FA group subjects with TT genotype showed a significantly greater homocysteine-lowering response than did subjects with CC genotype at week 4 or week 8, with or without adjustments for the relevant demographic and clinical characteristics at baseline (mean percent reduction of homocysteine at week 4: CC 5.9% vs. TT: 20.3%, *P *< 0.001; mean percent reduction of homocysteine at week 8: CC 10.8% vs. TT: 22.0%, *P *= 0.005). This heightened level of response was not shown in the low FA group (mean percent reduction of homocysteine at week 4: CC 8.8% vs. TT 10.6%, *P *= 0.769; mean percent reduction of homocysteine at week 8: CC 9.9% vs. TT 11.2%, *P *= 0.989) (Table 2).

The change in homocysteine was positively related to the baseline homocysteine concentrations in all genotypes and FA dosage groups. When baseline homocysteine also was adjusted, subjects with TT genotype seemed to have an attenuated homocysteine lowering response compared to subjects with CC genotype (*P *= 0.036 at week 4, *P *= 0.002 at week 8) in the low FA group, but not in the high FA group (*P *= 0.449 at week 4, *P *= 0.414 at week 8). Our results did not substantially alter with further adjustment for baseline folate concentration and folate change after treatment.

Compared to baseline, after 4 or 8 weeks of FA treatment, decreases in plasma homocysteine concentrations were seen in all MTR A2756G genotypes and FA dosage groups, except in subjects with AG/GG genotype in the low FA group at week 4. However, neither the homocysteine concentration post-FA treatment nor the homocysteine-lowering response of FA supplementation was affected by MTR A2756G polymorphism (Table 3). There was no significant interactive effect between MTHFR C677T and MTR A2756G polymorphisms on the homocysteine-lowering efficacy of different doses of FA supplementation with or without adjustments for potential non-genetic confounding factors.

**Table 3:** Response of plasma Homocysteine to different doses and duration of folic acid supplementation by MTR A2756G genotypes

	N	Homocysteine, μmol/L^1^ Percent relative change of homocysteine^2^			Difference in change of Homocysteine at 4 weeks by genotypes*^3^*			Difference in change of Homocysteine at 8 weeks by genotypes*^3^*		
		baseline	4 weeks	8 weeks	*β*	*SE*	*P*	*β*	*SE*	*P*
Control										
AA	130	12.8(1.6)[12.0]	12.9(1.6)[11.7]	12.9(1.6)[12.0]						
			1.7(15.1)	2.33(17.7)	ref			ref		.
AG/GG	22	12.1(1.5)[11.7]	12.6(6.8)[1.6]	12.6(1.5)[12.2]						
			5.8(15.2)	6.32(20.1)	5.17	3.50	0.139	6.08	4.10	0.138
Total	152	12.7(1.6)[12.0]	12.8(1.6)[11.7]	12.8(1.6)[12.0]						
			2.3(15.1)	2.9(18.0)						
Low FA										
AA	116	12.9(1.6)[11.5]	11.5(1.5)[11.1]*	11.3(1.5)[10.6] *						
			-9.4(16.5)	-10.2(19.4)	ref			ref		
AG/GG	31	14.1(1.5)[14.5]	13.1(1.5)[12.3]	12.5(1.5)[12.3] *^$^						
			-5.4 (15.9)	-9.6(18.3)	3.73	3.33	0.262	-0.06	3.86	0.989
Total	147	13.2(1.6)[11.6]	11.8(1.5)[11.3] *	11.6(1.5)[10.8] *^$%^						
			-8.6(16.4) ^%^	-10.1(19.1) ^%^						
High FA										
AA	119	13.7(1.6)[12.7]	11.8(1.5)[11.4] *	11.5(1.4)[10.8] *^$^						
			-12.3(16.2)	-14.4(17.3)	ref			ref		
AG/GG	27	13.5(1.8)[12.9]	11.6(1.5)[11.4] *	11.5(1.5)[10.7] *						
			-11.5(22.7)	-11.9(21.5)	1.30	3.39	0.700	2.72	3.60	0.450
Total	146	13.7(1.6)[12.7]	11.7(1.5)[11.4] *	11.5(1.5)[10.8] *^%^						
			-12.1(17.5) ^%^	-13.9(18.1) ^%^						

*^1^*Geometric mean (anti-log SD), median in brackets;

*^2^*[(Homocysteine after treatment - homocysteine at baseline)/( homocysteine at baseline)]*100, Mean(SD);

*^3^*Regression model was adjusted for age, sex, BMI, baseline SBP, DBP, creatinine, TG, HDL, TC, FPG, MTHFR C677T polymorphism and study centers;

*Significantly different from baseline, *P *< 0.05; ^$^Significantly different from week 4, *P *< 0.05;

^%^Significantly different from control group, *P *< 0.05.

We obtained similar results when we restricted our analyses to subjects who fully complied with the protocol (consuming at least 80% of all of the prescribed drugs) during the treatment periods (per protocol set, n = 381) (data not shown).

## Discussion

Both a previous dose-finding trial [10] and a dose-response trial [11] have discussed the relationship between homocysteine-lowering efficacy and optimal dose of FA supplementation, but without considering the possible modifying effect of MTHFR C677T polymorphism. A more recent study [12] evaluated the effect of MTHFR C677T polymorphism on the response to different doses of FA supplementation (0.1 mg/d, 0.4 mg/d, 4 mg/d and 4 mg/week) in northern Chinese women of childbearing age and reported that the MTHFR 677 TT genotype was an independent predictor of plasma homocysteine concentrations, irrespective of FA dose, which was consistent with our results. However, the study population consisted of young women and the FA dosage category was not optimal, being either too low (0.1 mg/d) or too high (4 mg/d). Furthermore, plasma homocysteine concentrations (7.2 μmol/L) in the study population (without FA fortification) were lower than those observed in a U.S. population [13] and a Chinese population [14], which means that this Chinese population was not a representative population in which to evaluate the homocysteine lowering effect of FA. In our study, we investigated if the change in plasma homocysteine concentration in response to two commonly used physiological doses (0.4 mg/d and 0.8 mg/d) of FA supplementation could be modified by individual MTHFR C677T and MTG A2756G polymorphisms in a multicenter, randomized, double-blind, controlled trial in hypertensive Chinese adults, whose homocysteine concentrations were comparable to the general Chinese population [14].

Consistent with a previous study [15], baseline homocysteine concentration was the major determinant of the homocysteine-lowering effect in all genotypes and FA dosage groups. Subsequently, in the present study, after 4 or 8 weeks of treatment, subjects with MTHFR 677 TT genotype had a significantly greater homocysteine-lowering response than did subjects with CC genotype in the high FA group. However, such a heightened response was not shown in the low FA group. Furthermore, when we further adjusted baseline homocysteine, subjects with TT genotype seemed to have a more attenuated homocysteine-lowering response than did subjects with CC genotype in the low FA group but not in the high FA group. The molecular mechanisms that underlie clinical remediation of enzyme variants are the ability of elevated levels of the cofactor or substrate to overcome the binding defects of Km mutants or to serve as a chemical chaperone to improve the stability of mutant enzyme variants [16,17]. Our results further suggest that daily 0.4 mg FA supplementation may be insufficient, and that a daily dose of 0.8 mg FA may be required to lower homocysteine concentration in subjects with TT genotype.

Furthermore, after the treatment, subjects with CT genotype had significantly higher homocysteine concentrations than those with CC genotype in the low FA group, and had similar homocysteine concentrations as CC subjects in the high FA group. These results indicate that daily 0.8 mg FA also may be necessary for lowering homocysteine concentration in subjects with CT genotypes. However, although they showed a greater homocysteine lowering effect than subjects with CC genotype, subjects with TT genotype still had the highest homocysteine concentrations even after 8 weeks of high FA treatment. Therefore, other therapeutic strategies need to be developed to reduce the risk associated with hyperhomocysteinemia in TT subjects.

In fact, Malinow et al. [18] first reported that TT subjects experienced much greater decreases in plasma total homocysteine concentrations after receiving FA at a dose of 1 or 2 mg/d for 3 weeks than did CC subjects. Among subjects who were not previously taking multivitamins, the mean reductions in plasma total homocysteine concentrations were -20.9%, -13.1%, and -7.1% in persons with the TT, CT, and CC genotypes, respectively (*P *= 0.019 for TT versus CC). A similar result also has been reported in a study in Taiwan, in which 5 mg of FA daily were supplemented for 8 wks [19]. Our results appear to be consistent with these studies. However, the study of Woodside et al. [20] showed that TT subjects are less responsive to the effects of FA and B-vitamin supplementation (daily 1 mg FA, 7.2 mg Vitamin B6, and 0.02 mg Vitamin B12 for 8 wks.) than CC subjects. And Ho GY et al. [21] also reported that MTHFR C677T did not impact the total homocysteine-lowering effect of vitamins (daily 2.5 mg FA, 25 mg Vitamin B6, and 0.5 mg Vitamin B12 for 1 year) in a study performed in Singapore. The reasons for the disparities among the results of these studies are unknown. Additional large dose-finding studies are required to further elucidate the possible contributory factors, such as folate status, ethnicity of the participants, and in particular the possible effects of concomitant vitamin B6 and/or B12.

Our study showed that neither homocysteine concentration at baseline or post-FA treatment nor the homocysteine lowering response of FA supplementation was affected by MTR A2756G polymorphism. However, there was a significant homocysteine reduction in MTR 2756 AG/GG genotype after 4 weeks of FA supplementation in the high FA (0.8 mg/d) group, but not in the low FA (0.4 mg/d) group, which means that we cannot exclude the possible role of MTR A2756G polymorphism in the homocysteine metabolic pathway.

Overall, our results suggest that daily 0.8 mg FA may be necessary to lower homocysteine concentration for Chinese hypertensive subjects with CT or TT genotype, which have important clinical and public health implications. Increased homocysteine concentration has been implicated as a risk factor for neural tube defects (NTDs) and Downs syndrome [22,23]. A study by Wilcken et al. reported that the frequency of CT and TT genotypes in Hungary was about 45% and 11.1%, respectively [24]. Two Hungarian intervention trials [25,26], which used a multivitamin containing 0.8 mg folic acid, found a significant reduction in the first occurrence of urinary tract and cardiovascular abnormalities beyond the 90% reduction of NTDs. There is no evidence that 0.4 mg folic acid has a similar preventive effect on defects beyond NTDs. Our results further suggest that there may be an additional benefit if a daily dose of 0.8 mg, but not 0.4 mg, of folic acid could be used in women, particularly in populations with a high frequency of T allele (such as the Chinese) and without folic acid fortification. Furthermore, identifying TT or CT subjects and providing them with an optimal dose of folic acid also may be very important in the prevention of CVD [4,27] in populations without folic acid fortification.

Our study has the following strengths. This was a randomized, multicenter and double blind trial. This study simultaneously assessed the effect of FA dosages and gene polymorphisms on homocysteine-lowering efficacy. Our trial studied two commonly used doses of FA, which are of important clinical and public health relevance. However, caution is needed in generalizing our findings from this hypertensive Chinese population to other populations. Furthermore, though similar results were obtained when we restricted our analyses to subjects who fully complied with the protocol during the treatment periods, we still could not fully exclude the effect of non-compliance problem on our results. Also, it should be noted that we did not measure other polymorphisms in the folate pathway, such as the MTHFR A1298C variant. Furthermore, the treatment period with FA was only 8 weeks and the sample size was rather small when sub-grouped into various groups (control, low FA and high FA) for genetic association studies. Future studies with longer treatment duration and a larger sample size are needed to confirm our results. The association between angiotensin-converting enzyme inhibitor treatment and the change of homocysteine was controversial [28,29]. We did not observe a significant relationship between enalapril treatment and homocysteine change in our study.. Additional large sample studies also are needed to further examine the relationship of enalapril treatment with homocysteine change and the possible interactive effects between enalapril and folic acid on the change in homocysteine.

## Conclusions

We demonstrated that MTHFR C677T polymorphisms can not only affect plasma homocysteine levels at baseline and post-FA treatment, but also modify therapeutic response to various dosages of FA supplementation. These findings, if confirmed by additional studies, may enable medical providers to offer more personalized care and advice on FA supplementation for a given individual.

## List of abbreviations

CI: Confidence Interval; CVD: cardiovascular disease; FA: folic acid; MTHFR: methylenetetrahydrofolate reductase; MTR: methionine synthase; RR: Relative Risk.

## Competing interests

The authors declare that they have no competing interests.

## Authors' contributions

QX, LJ, CY, LZ, ZZ, GJ, GD, HJ, WY, ZF, XX, WX, XX and HY participated in the design of the study; LJ, CY, LZ, GJ, GD, HJ, WY, ZF, HY conducted the study; QX, LJ, XXu, WX, XXu and YH analyzed data; QX, LJ, CY, LZ, ZZ, GJ, GD, HJ, WY, ZF, XX, WX, XX and HY wrote the paper; all authors read and approved the final manuscript.
